# Commentary: Organ ARCs: Assessment and repair centers in transplantation as gateways to meaningful therapeutic interventions

**DOI:** 10.1016/j.xjon.2020.08.004

**Published:** 2020-08-08

**Authors:** Bryan A. Whitson, Sylvester M. Black

**Affiliations:** a Division of Cardiac Surgery, Department of Surgery, The Ohio State University Wexner Medical Center, Columbus, Ohio; b The Collaboration for Organ Perfusion Protection and Regeneration (COPPER) Laboratory, The Ohio State University Wexner Medical Center, Columbus, Ohio; c Division of Transplant, Department of Surgery, The Ohio State University Wexner Medical Center, Columbus, Ohio

**Figure:**
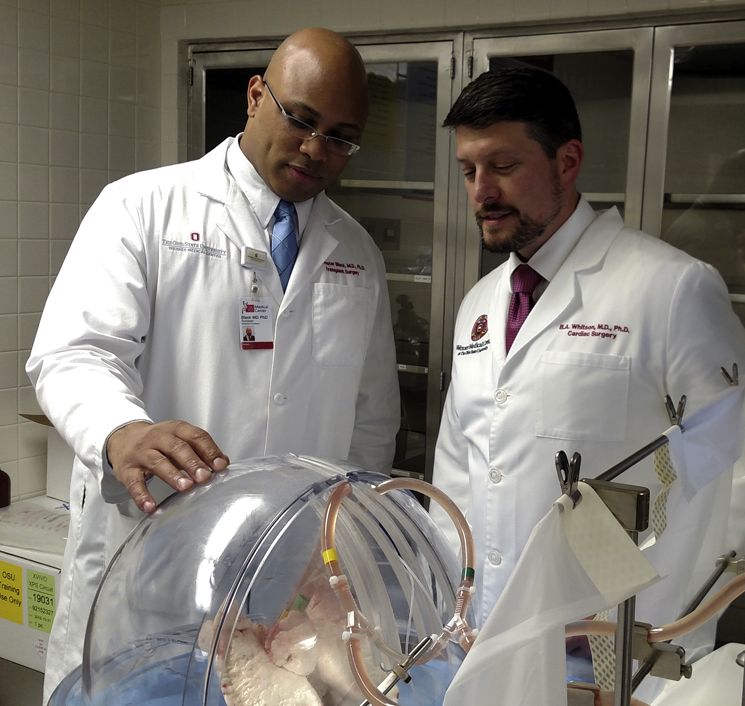
Drs Whitson and Black in The Ohio State University Organ ARC.

**Central Message**

Normothermic ex vivo organ perfusions enable the creation of organ assessment and repair centers (organ ARCs), which allows for improved donor organ quality and expanding donor organ availability.

See Article page 164.

In 2001, the world was shown the future of solid-organ transplantation with the successful ex vivo lung perfusion (EVLP) and transplantation performed by Steen and colleagues from Lund University Hospital, Lund, Sweden.^1^ Perhaps at the time, the revolutionary nature of the accomplishment was underappreciated, although it set into motion a vision that Dr Alexis Carrel and Charles Lindbergh had when they published their foreshadowing book *The Culture of Organs* in 1938.^2^ From what may have been perceived as Shelleyian science fiction, the University of Toronto Lung Transplant Program under the leadership of Dr Keshavjee has made a reality.^3^^,^^4^

As with a great many of the pivotal advances in lung transplantation attained at the University of Toronto, the refinement and operationalization of a robust, cross-continent lung repair center using normothermic machine perfusion has had a profound impact on their recipients lives but also on the whole field of transplantation. In this month's edition of the *Journal*, Keshavjee^4^ provides us with the history of the evolution of the Toronto EVLP program and their realization of Organ Repair Centers. While the Toronto Lung Transplant Program has brought lung assessment and repair into the mainstream, the implications of normothermic ex vivo organ perfusion (NEVOP) on the field of transplantation as a whole has fundamentally been changed.

With the conceptualization and realization of NEVOP, 2 fundamental limitations of transplantation have been overcome: first, with NEVOP, time becomes somewhat relative. Second, with NEVOP, we have the ability to repair donor organs once thought to be marginal, extended criteria, or poor quality. When we are able to maintain organs and ensure viability with NEVOP, whether via blood-based or acellular perfusates, we make ischemic time relative. This enables the transport of organs across vast distances. Removing time and distance as barriers allows for better donor–recipient matching, both size and immunologically, both of these contribute to improved outcomes. As we are able to ensure organ viability over time, we have the ability to assess the organ for adequate quality, both physiologically and with molecular biology. This combination with NEVOP lets us create true organ assessment and repair centers, or organ assessment and repair centers,^5^ not only for lungs but hearts, livers, kidneys, and potentially even pancreas, intestines, or vascular composite allografts. With the NEVOP platform, one has the ability to deliver nanoparticle or viral vectors,^6^ modify genes,^7^ or even scaffold organs.^8^ This would allow for truly personalized medicine by tailoring the donor organ to each recipient.

Another perspective to normothermic perfusion and organ repair, which Dr Keshavjee refers to, is the commercialization of organ repair centers. Conceptually, there are benefits to reproducibility and focused expertise, although one would be a little cautious of commercializing organ transplantation. Done thoughtfully, the types of services provided by Lung Bioengineering would extend the benefits of NEVOP to those transplant centers that do not have the infrastructure to do NEVOP in house.

As with so many of the advances in lung transplantation, the University of Toronto Lung Transplant Program has led the way in normothermic EVLP and will continue to do so. These advances will lead us into a new era of NEVOP and organ assessment and repair centers to expand the benefit of transplantation to more patients with improved outcomes.
